# Effectiveness of household lockable pesticide storage to reduce pesticide self-poisoning in rural Asia: a community-based, cluster-randomised controlled trial

**DOI:** 10.1016/S0140-6736(17)31961-X

**Published:** 2017-10-21

**Authors:** Melissa Pearson, Chris Metcalfe, Shaluka Jayamanne, David Gunnell, Manjula Weerasinghe, Ravi Pieris, Chamil Priyadarshana, Duleeka W Knipe, Keith Hawton, Andrew H Dawson, Palitha Bandara, Dhammika deSilva, Indika Gawarammana, Michael Eddleston, Flemming Konradsen

**Affiliations:** a Pharmacology, Toxicology and Therapeutics, University/BHF Centre for Cardiovascular Science, and Centre for Pesticide Suicide Prevention, University of Edinburgh, Edinburgh, UK; b South Asian Clinical Toxicology Research Collaboration (SACTRC), Faculty of Medicine, University of Peradeniya, Peradeniya, Sri Lanka; c School of Social and Community Medicine, University of Bristol, Bristol, UK; d Faculty of Medicine, University of Kelaniya, Kelaniya, Sri Lanka; e Department of Community Medicine, Rajarata University of Sri Lanka, Anuradhapura, Sri Lanka; f Centre for Suicide Research, Department of Psychiatry, University of Oxford, Oxford, UK; g Sydney Medical School, University of Sydney, Sydney, Australia; h Provincial Department of Health Services, Anuradhapura, North Central Province, Sri Lanka; i Department of Medicine, Faculty of Medicine, University of Peradeniya, Peradeniya, Sri Lanka; j Department of Public Health, Faculty of Health and Medical Sciences, University of Copenhagen, Copenhagen, Denmark

## Abstract

**Background:**

Agricultural pesticide self-poisoning is a major public health problem in rural Asia. The use of safer household pesticide storage has been promoted to prevent deaths, but there is no evidence of effectiveness. We aimed to test the effectiveness of lockable household containers for prevention of pesticide self-poisoning.

**Methods:**

We did a community-based, cluster-randomised controlled trial in a rural area of North Central Province, Sri Lanka. Clusters of households were randomly assigned (1:1), with a sequence computer-generated by a minimisation process, to intervention or usual practice (control) groups. Intervention households that had farmed or had used or stored pesticide in the preceding agricultural season were given a lockable storage container. Further promotion of use of the containers was restricted to community posters and 6-monthly reminders during routine community meetings. The primary outcome was incidence of pesticide self-poisoning in people aged 14 years or older during 3 years of follow-up. Identification of outcome events was done by staff who were unaware of group allocation. Analysis was by intention to treat. This trial is registered with ClinicalTrials.gov, number NCT1146496.

**Findings:**

Between Dec 31, 2010, and Feb 2, 2013, we randomly assigned 90 rural villages to the intervention group and 90 to the control group. 27 091 households (114 168 individuals) in the intervention group and 26 291 households (109 693 individuals) in the control group consented to participate. 20 457 household pesticide storage containers were distributed. In individuals aged 14 years or older, 611 cases of pesticide self-poisoning had occurred by 3 years in the intervention group compared with 641 cases in the control group; incidence of pesticide self-poisoning did not differ between groups (293·3 per 100 000 person-years of follow-up in the intervention group *vs* 318·0 per 100 000 in the control group; rate ratio [RR] 0·93, 95% CI 0·80–1·08; p=0·33). We found no evidence of switching from pesticide self-poisoning to other forms of self-harm, with no significant difference in the number of fatal (82 in the intervention group *vs* 67 in the control group; RR 1·22, 0·88–1·68]) or non-fatal (1135 *vs* 1153; RR 0·97, 0·86–1·08) self-harm events involving all methods.

**Interpretation:**

We found no evidence that means reduction through improved household pesticide storage reduces pesticide self-poisoning. Other approaches, particularly removal of highly hazardous pesticides from agricultural practice, are likely to be more effective for suicide prevention in rural Asia.

**Funding:**

Wellcome Trust, with additional support from the American Foundation for Suicide Prevention, Lister Institute of Preventive Medicine, Chief Scientist Office of Scotland, University of Copenhagen, and NHMRC Australia.

## Introduction

Pesticide self-poisoning is a major public health problem in rural Asia^1^, ^2^ and a substantial burden on health services.^3^ A systematic review^4^ of data from 2006–15 showed that an estimated 89% of all global suicides from pesticide self-poisoning occurred in the Asian and Western Pacific regions. WHO recognises pesticide ingestion to be one of the three most important means of suicide worldwide.^2^, ^4^ In Sri Lanka, self-poisoning with pesticides is the most common method of self-harm in many rural districts,^5^ highly lethal,^6^ associated with impulsivity,^7^, ^8^, ^9^ and the fifth leading cause of death in 2012.^10^

Means restriction is a key element of suicide prevention strategies.^11^, ^12^, ^13^ Restricting access to common and highly lethal methods of suicide can reduce both method-specific and all-cause suicide rates.^14^, ^15^, ^16^ Such approaches for pesticide self-poisoning include administrative interventions altering behaviour (particularly the purchase, use, and storage of pesticides) and interventions altering the availability of highly hazardous pesticides in the community (through regulatory action to remove such pesticides from agricultural practice).^17^ Interventions working at the patient level, to improve provision of medical care in resource-poor hospitals, are difficult for the most common pesticides used for suicide (organophosphorus and carbamate insecticides, and paraquat);^18^, ^19^ it is unlikely that improved care will be a highly effective approach to suicide prevention.

**Research in context**

**Evidence before this study**

We searched PubMed for articles published between Jan 1, 1990, and May 1, 2017, with the search terms (((“pesticide”) AND “storage”) AND “intervention”). 12 references were retrieved but none reported randomised controlled trials or systematic reviews. A further search of the internet using general search engines with keywords “safe storage” AND “intervention” identified pilot studies of pesticide storage in Sri Lanka, China, and India, as well as the WHO report on safer access to pesticides for suicide prevention. These studies highlighted the potential for improved storage on the basis of the acceptability of such devices in the community. Additional searches of “pesticide” AND “poisoning” identified four randomised controlled studies that focused on clinical management of poisoning. To our knowledge, no effectiveness studies of pesticide storage to prevent pesticide poisoning have been done.

**Added value of this study**

This study is the first effectiveness trial of improved pesticide storage to prevent pesticide poisoning. The provision of a lockable storage container to householders was designed through discussion and pilot studies to be robust and acceptable to farming communities. Our study tested the effectiveness of pesticide storage at a population level to determine if this intervention could make a significant contribution to reducing pesticide poisoning in rural Asian communities.

**Implications of all the available evidence**

The results of our study show that improved pesticide storage in households is not an effective intervention to prevent pesticide self-poisoning, despite its community acceptability. Our research counters the current policy approaches advocating improved storage of pesticides to reduce intentional pesticide poisoning. Only withdrawal of the most highly hazardous pesticides from agricultural practice has been shown to reduce deaths from pesticide poisoning. Global public health efforts should focus on this approach to rapidly reduce pesticide suicides worldwide.

WHO, the pesticide industry, and the International Association for Suicide Prevention (IASP) have advocated the use of improved household and community storage, with locked boxes or lockers, to prevent pesticide self-poisoning as part of an overall suicide prevention strategy, termed “safer storage”.^20^ Findings from pilot studies of improved household storage in Sri Lanka^21^, ^22^ and China^20^ and studies of community lockers in India^23^ suggest that the approach is appreciated by farming communities. However, the trials were pilot in nature and not designed to assess effectiveness; additionally, repeated interaction with the communities to assess use of the storage devices might have affected their utilisation.

Domestic locked boxes can result in pesticides being brought into the home from the field where they are often stored, potentially increasing the risk of self-poisoning. This problem is exacerbated because locking of boxes reduces over time; households might also find it difficult to keep the key hidden from vulnerable household members.^21^, ^24^ Real world use of community lockers is uncertain because they often require farmers to walk away from their fields towards the store in the centre of the village, and a second person to be present for the locker to be opened.^23^

In view of the paucity of evidence for effectiveness of safer storage of pesticides and the potential for increased risk of harm, we aimed to test the effectiveness of household pesticide storage containers in a large community-based, cluster-randomised controlled trial.

## Methods

### Study design and participants

**Figure 1:**
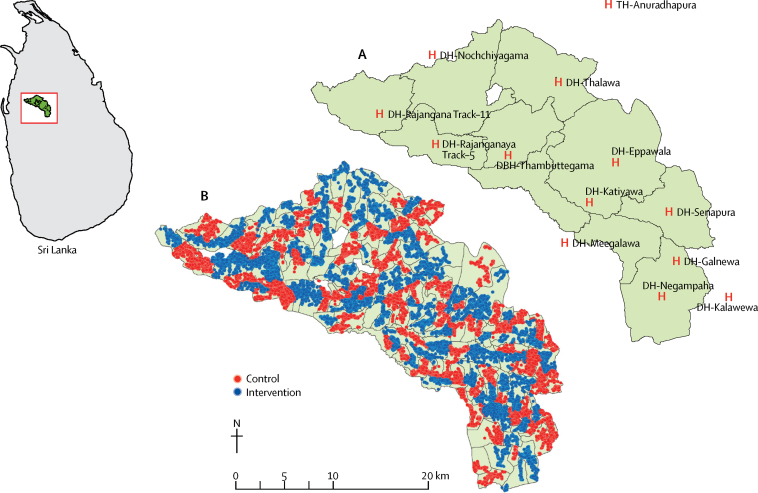
Location of the study area in the North Central Province of Sri Lanka (A) Distribution of households in the study area by study group. Date June 22, 2017. (B) Hospitals used to identify outcome events. Each dot represents a household. Author: Safe Storage Study team. DH=district hospital. DBH=district base hospital. TH=teaching hospital.

We did a community-based, cluster-randomised controlled trial of a lockable pesticide storage container in a rural area of Sri Lanka. A description of the study design and methods has been published elsewhere.^25^ The study was done in the Anuradhapura District (population 855 562, census 2011) of Sri Lanka's North Central Province (figure 1). We recruited geographical clusters of households primarily from the Mahaweli H irrigation region, including the divisional secretariats of Thambuttegama, Thalawa, Galnewa, Rajanganaya, Ipolagama, and Nochchiyagama, because of the high use of pesticides in agriculture and high incidence of pesticide self-poisoning in this region. All communities within the study area were eligible for participation apart from those recruited to our previous pilot studies (1026 households).^22^, ^24^ The chief village official (*Grama Niladhari*) was approached to seek consent for community enrolment; individual household verbal consent was then sought at the start of each household survey.

At enrolment, adult householders present in the home were interviewed about household sociodemographic information, current pesticide use and storage practices, and previous history of self-harm and alcohol consumption. Household global positioning system (GPS) coordinates were recorded with a Juno device (Trimble Inc, USA).^26^ The questionnaire was administered by young adults, mostly from and familiar with the local area, in the local language, after training and with regular audit. Additional details of this interview process and quality control have been published elsewhere.^25^, ^27^

There were no substantial changes to the protocol after the trial started. We initially planned to collect case data from both village level public health midwives and small rural peripheral hospitals. However, early pilot work, before the distribution of lockable containers, showed the difficulties of midwives as sources of case data and additional resources were deployed at peripheral hospital units. The results of the end of study demographic survey showed this strategy to be highly effective (see discussion). Another change to the protocol was a reduction in the proportion of the study sample that received the end of study demographic survey from 100% to 26%, for logistical reasons.

Ethics approval was received from the research ethics committees of the University of Peradeniya and Rajarata University of Sri Lanka. The study was approved by the Provincial Department of Health Services, North Central Province, and the Sri Lankan Ministry of Health. A data monitoring committee was established for the trial and a charter written. No formal stopping rules or interim analyses were planned. The data monitoring committee was responsible for safeguarding the interests of trial participants and monitoring the quality of the research.

### Randomisation and masking

Clusters of households were the unit of randomisation for this study (median number of households per cluster 272 [IQR 207–344]). For logistical reasons, clusters were grouped into ten bands. Clusters were delineated after completion of household surveys in each band. Cluster boundaries were not based on civil village boundaries alone because some villages were closely intertwined, increasing the risk of contamination. To increase geographical separation, and reduce the risk of contamination between clusters caused by, for example, the onward sale of unused storage containers, we actively identified natural and social boundaries between communities. This approach included identifying patterns of social interaction between communities, geographical features such as irrigation canals, roads, and forests, as well as means of and reasons for access such as transport to commercial areas, schools, and temples. Random allocation of clusters within each band to the intervention or control groups was done by a person not involved in recruitment, intervention, or monitoring, with a bespoke computer program written in Stata. Minimisation was used to reduce imbalance between groups in the number of clusters allocated to each group, number of individuals in households eligible for a pesticide storage container, and rate of previous pesticide self-poisoning in the cluster. A random aspect was maintained in the minimisation, which prevented allocations being anticipated.^25^

To maintain masking and minimise bias, we had separate study teams for recruitment, randomisation, intervention, outcome data collection, outcome linkage, and analysis, with limited interaction.^25^ Identification of outcome events was done by staff who were unaware of group allocation.

### Procedures

After randomisation, the *Grama Niladharis* in intervention clusters were informed and arrangements made for the distribution of lockable pesticide storage containers to eligible households. Households were deemed eligible if they farmed or had used or stored pesticides in the preceding agricultural season. To encourage compliance and reduce the risks associated with bringing pesticides into the home, we took care to design a container acceptable to the community^24^, ^25^ that could be positioned outside the house. The design of the lockable storage container was informed by discussion with the local communities and 4 years of piloting.^24^ The container was made from ultraviolet-resistant plastic (appendix); we recommended that it be buried in the ground for security.^25^ The container had two lids to protect the lock and the contents, respectively, against moisture. Each container had 20 cm anchors extending sideways from the bottom to prevent it being pulled up out of the ground. A small community demonstration was given to recipients to promote the correct installation and use of the container. Substantial efforts were made to ensure that containers were installed; farmers were given a choice as to their preferred location for the container—ie, in their fields, home garden, or house. Researchers subsequently visited households to ensure installation in all eligible households. Further promotion of use of the containers after distribution was restricted to posters hung up in intervention communities and 6-monthly presentations at community farmer meetings. No contact was made with households for 3 years, apart from those in the five sub-villages (605 households, 2% of intervention households) randomly chosen from intervention clusters to study use of the lockable containers. Households in the control group received no intervention.

Data on cases of fatal and non-fatal pesticide self-poisoning, accidental poisoning, and all forms of non-fatal and fatal self-harm were prospectively collected from several sources. Most patients with poisoning or self-harm presented first to small peripheral hospitals (median number of beds 42 [range 12–133]) spread across the district (figure 1). After triage and treatment, some patients were transferred to a secondary level hospital within the study area (Thambuttegama) or the main Anuradhapura District tertiary level hospital (Anuradhapura Teaching Hospital). Patients admitted for poisoning or self-harm to the two main hospitals were identified by research assistants attending the medical wards daily, and checking admissions to surgical, paediatric, and intensive care wards on a weekly basis, and the Anuradhapura Teaching Hospital morgue at the end of follow-up.

All remaining peripheral hospitals (n=9) within the study area were visited at least every other day by researchers to identify poisoning or self-harm admissions. Cases were identified by researchers who were not aware of the person's allocation. Field research officers also visited hospitals situated just outside the study area (n=2, figure 1) to identify cases that had bypassed the local hospital, through checks of the admission and transfer books and discussion with relevant staff. During the study, we built up close relationships with medical and nursing staff in the peripheral hospitals, resulting in researchers often being telephoned when cases presented to these hospitals. Private inpatient care is restricted in the district; our previous surveys of these hospitals suggested that patients who self-harm did not seek care in these facilities.^28^

Deaths that occurred before hospital presentation were identified by regular review of the records of local coroners and police. At the end of the study, we sought additional cases by examination of records belonging to local magistrates and district coroners.

After identification of a case, a researcher obtained demographic and address data from the medical records, the patient, or a relative. The researcher did not enquire about the presence of pesticide containers in the case's household to sustain masking to allocation. These data were shared by telephone with a case-matching researcher in a central office who searched for the person on the baseline survey database and allocated the case to a unique household or individual study identifier. If necessary, the researcher re-contacted the patient or relative in hospital, or rarely in their village if the patient had been discharged, to obtain further information to allow matching to baseline records. Again, no effort was made to identify whether the household had a pesticide storage container. The case-matching researcher had no role in randomisation, container distribution, case follow-up, or data collection.

Follow-up to identify study endpoints was started after the first round of container distribution in each band and continued until 3 years after the last round of distribution. After completion of 3 years of follow-up, a repeat household survey was done in 13 999 (26%) households to estimate migration in and out of the area during the study, and to assess the use and locking of pesticide containers.

To assess adherence during the 3 years of follow-up, five sub-villages in five intervention clusters were selected at random from across the study area for annual review of use of the pesticide containers. Each village was visited twice during the study, use of containers recorded, and household opinions on their usefulness elicited through a survey and focus groups.

### Outcomes

The primary outcome was the incidence of pesticide self-poisoning, whether fatal or non-fatal, in individuals aged 14 years or older, during a 3-year follow-up period starting from completion of container distribution to each band (follow-up was staggered across bands). Secondary outcomes were the incidence of pesticide poisoning in children (aged <14 years), pesticide poisoning in general (deliberate and accidental, all ages), self-poisoning (all substances, fatal and non-fatal, age ≥14 years), non-fatal self-harm (all methods, age ≥14 years), fatal self-harm (all methods, age ≥14 years), non-fatal non-pesticide self-poisoning (age ≥14 years), and fatal non-pesticide self-poisoning (age ≥14 years).

### Statistical analysis

We have previously justified the sample size target in detail.^25^ Assuming a primary outcome incidence of 175 per 100 000 person-years in the control group, and an inflation factor of 1·75 to accommodate the clustered design, further accommodating contamination and non-use of safe storage containers, at least 217 944 person-years of follow-up in each study group (approximately 24 216 households; 81 clusters per group; 162 in total) would give 80% power to detect a 33% reduction to 117 events per 100 000 person-years in the intervention group.

The main analysis was prespecified in a signed and dated (Aug 23, 2016) statistical analysis plan that was made publicly available before release of the data for analysis. The primary analysis followed the intention-to-treat principle, comparing the observed incidence of self-poisoning with pesticides between individuals in clusters allocated to the intervention group, and individuals in clusters allocated to the control group. A random effect Poisson regression model was used, accommodating variation between clusters in the primary outcome incidence as a gamma distribution. This analysis was adjusted for the minimisation variables—ie, number of individuals in households eligible for a lockable storage container and rate of previous pesticide self-poisoning in the cluster, both included as a trend term across three tertiles. This approach was adapted to each of the secondary outcomes.

Prespecified subgroup analyses investigated whether the effectiveness of the intervention was modified by the cluster-level historical rate of self-poisoning, the cluster-level proportion of households reporting a member having problems with alcohol (both established in the baseline survey), the cluster-level proportion of households provided with a lockable box, and the year of follow-up. For each of these analyses in turn interaction terms were generated, distinguishing the subgroups in the intervention and control groups, and these were added to the statistical model to test the evidence that the intervention effect varied by subgroup. We did a sensitivity analysis excluding the five clusters in which container use was reviewed, because their participation in this assessment might have increased household compliance with safe storage. All analyses were done with Stata statistical software, version 14.2. This trial is registered with ClinicalTrials.gov, number NCT1146496.

### Role of the funding source

The funders of the study had no role in study design, data collection, data analysis, data interpretation, or writing of the report. The corresponding authors had full access to all the data in the study and had final responsibility for the decision to submit for publication.

## Results

**Figure 2:**
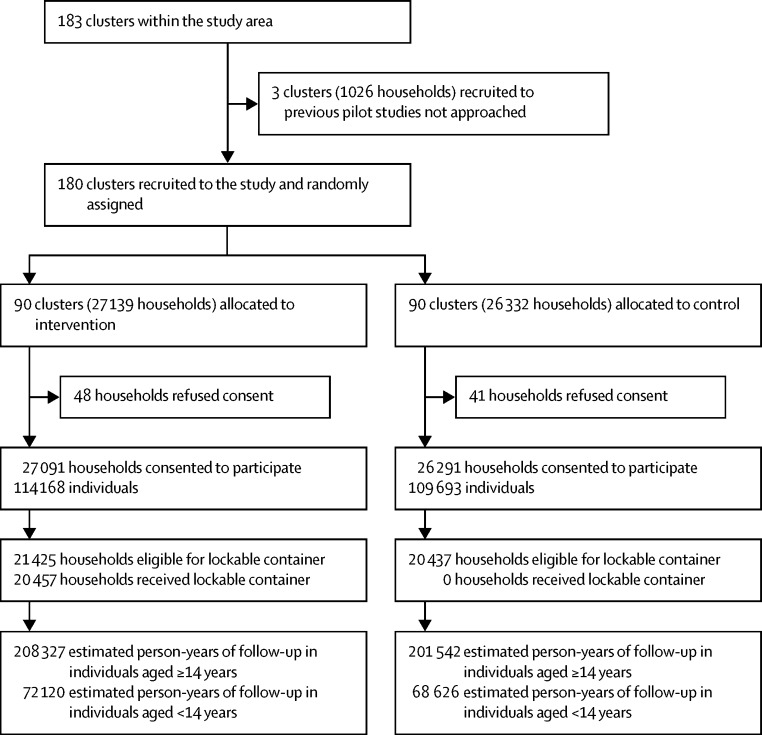
Trial profile

Between Dec 31, 2010, and Feb 2, 2013, we enrolled 180 clusters, of which 90 were randomly assigned to the intervention group and 90 to the control group (figure 2). Follow-up started on July 29, 2011, with distribution of lockable pesticide storage containers to the first band, and finished on May 12, 2016, 3 years after distribution of containers to the tenth band.

53 382 households (comprising 223 861 individuals) gave consent to participate. Measures of socioeconomic status, number of households, pesticide use, and reported history of pesticide self-harm and alcohol use were well balanced between the two groups (table 1). 87 751 individuals aged 14 years or older in the intervention group and 84 469 in the control group were followed up for cases. During the 3 years of the trial, migration was modest with an estimated 5% (1570 of 29 355) and 6% (1668 of 29 670) of the population migrating in, and 11% (3168 of 29 355) and 11% (3352 of 29 670) of the population migrating away, in the intervention and control areas, respectively.

**Table 1:** Baseline characteristics of clusters

		**Intervention**	**Control**
**Clusters**			
Number of clusters		90	90
Number of consenting households*		27 091	26 291
Households eligible for a lockable box		21 425 (79%)	20 437 (78%)
Households eligible for a lockable box and receiving one		20 457 (95%)	0
Households reporting a previous case of pesticide self-harm		2518 (9%)	2466 (9%)
Households reporting a member with a problem with alcohol use		6851 (25%)	6660 (25%)
Household construction			
	Solid construction, durable materials	12 715 (47%)	12 443 (47%)
	Semi-permanent construction, mixture of materials	11 875 (44%)	11 375 (43%)
	Improvised construction, non-durable materials	2474 (9%)	2443 (9%)
	Unknown	27 (<1%)	30 (<1%)
Household possession of motorised vehicle			
	Four wheels (car, tractor)	2088 (8%)	2075 (8%)
	Two to three wheels (motorbike)	14 996 (55%)	14 363 (55%)
**Individuals**			
Number of individuals		114 168	109 693
Number of individuals aged ≥14 years		87 751	84 469
Number of female individuals aged ≥14 years		44 693	43 105
Age (years)		31·4 (19·8)	31·5 (19·8)
Individuals aged ≥14 years in households eligible for a lockable box, and resident there			
	All year round	46 120 (65%)	44 239 (65%)
	7–11 months	9950 (14%)	9461 (14%)
	1–6 months	12 058 (17%)	11 164 (17%)
	<30 days	2908 (4%)	2772 (4%)

Data are n (%) or mean (SD), unless otherwise stated.

* 48 households in the intervention group and 41 in the control group refused to take part in the baseline survey.

Pesticide storage containers were distributed to 20 457 households. At the 2-week check, 19 534 (95%) containers were installed within the home compound, 41 (<1%) within the home, and 738 (4%) in the field. The location of 144 (1%) containers was unknown or they had been returned. At the end of 3 years of follow-up, surveys of 6937 (26%) of 27 091 households in the intervention group showed that 4264 (61%) households were storing pesticides and 3698 (53%) were locking pesticides away sometimes or always. Surveys of 7062 (27%) of 26 291 households in the control group showed that a similar number (3681 [52%]) were storing pesticides but only 351 (5%) households were locking pesticides away sometimes or always. Data from 605 households in the five villages in which container use was assessed (number of households eligible for container 52, 78, 104, 171, and 208, respectively) showed that self-reported use of the locked containers was 72% (367 of 507) after 1 year and 76% (339 of 448) after 2 years.

We recorded 1252 cases of pesticide self-poisoning in individuals aged 14 years or older from the study area during the 3 years of follow-up. Of these 1252 cases, 1077 (86%) were matched to an individual participating in the baseline survey, 92 (7%) to households (either new members of the household or individuals missed in the baseline survey), and 83 (7%) were cluster matches (we were unable to match them exactly to a house, knowing only their cluster—and therefore allocation to intervention or control group). Of the cases matched to individuals, 516 cases occurred in 500 individuals in the intervention group (15 [3%] individuals had more than one episode), and 561 cases occurred in 547 individuals in the control group (12 [2%] individuals had more than one episode).

1931 records were collected for the 1252 cases of pesticide self-poisoning. Of these records, 1203 (62%) were identified in peripheral hospital records, 646 (34%) in Teaching Hospital records, and 82 (4%) in records from the police or coroner. We observed no difference between study groups in the source of data collection for cases. 11 deaths that occurred before hospital presentation were identified by review of the records of local coroners and seven by review of police records. 11 deaths that occurred before hospital presentation were identified in routine monitoring during the surveillance period and an additional six cases at the end of the 3 year follow-up.

There were fewer cases of pesticide self-poisoning in the intervention group (611 cases) than in the control group (641 cases; table 2); the incidence of pesticide self-poisoning in individuals aged 14 years or older did not differ between groups (293·3 per 100 000 person-years of follow-up in the intervention group *vs* 318·0 per 100 000 in the control group; rate ratio 0·93, 95% CI 0·80–1·08; p=0·33). In a sensitivity analysis, there was no effect of including or excluding the five villages in which there was greater researcher presence while use of the containers was assessed (appendix).

**Table 2:** Primary and secondary outcomes

	**Number of events**	**Person-years of follow-up**	**Incidence per 100 000 person-years**	Rate ratio*(95% CI)	**p value**†
**Primary outcome: pesticide self-poisoning (age ≥14 years)**					
Intervention	611	208 327	293·3	0·93 (0·80–1·08)	0·33
Control	641	201 542	318·0	··	··
**Pesticide poisoning in children (age <14 years)**					
Intervention	18	72 120	25·0	1·21 (0·58–2·55)	0·61
Control	15	68 626	21·8	··	··
**All pesticide poisoning, deliberate and accidental (all ages)**					
Intervention	633	280 635	225·6	0·93 (0·81–1·07)	0·31
Control	662	270 334	244·9	··	··
**Self-poisoning, all substances (age ≥14 years)**					
Intervention	1155	208 327	554·4	0·97 (0·86–1·08)	0·55
Control	1173	201 542	582·0	··	··
**Non-fatal self-harm, all methods (age ≥14 years)**					
Intervention	1135	208 327	544·8	0·97 (0·86–1·08)	0·56
Control	1153	201 542	572·1	··	··
**Fatal self-harm, fatal pesticide self-poisoning (age ≥14 years)**					
Intervention	47	208 327	22·6	1·22 (0·79–1·87)	0·37
Control	38	201 542	18·8	··	··
**Fatal self-harm, all methods (age ≥14 years)**					
Intervention	82	208 327	39·4	1·22 (0·88–1·68)	0·23
Control	67	201 542	33·2	··	··
**Non-pesticide non-fatal self-poisoning (age ≥14 years)**					
Intervention	540	208 327	259·2	1·01 (0·88–1·17)	0·86
Control	523	201 542	259·5	··	··
**Non-pesticide fatal self-poisoning (age ≥14 years)**					
Intervention	4	208 327	1·9	0·44 (0·13–1·55)	0·18
Control	9	201 542	4·5	··	··

288 individuals without an age recorded are not included in the age-specific outcome measures.

* The estimated rate ratio is adjusted for number of person-years of follow-up of people in households eligible for a lockable box, and rate of previous pesticide self-poisoning in the cluster.

† p values are from likelihood ratio tests.

**Figure 3:**
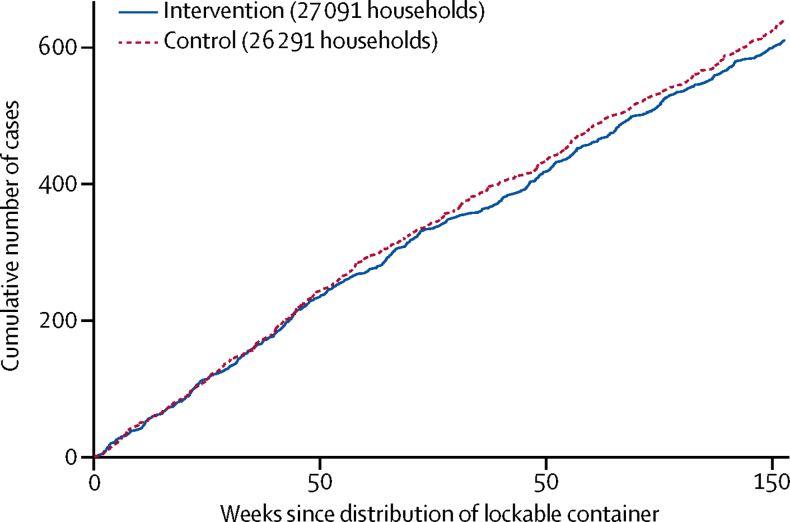
Cumulative number of cases of pesticide self-poisoning

We found no evidence that the effect of lockable container provision differed according to the historical incidence of pesticide self-poisoning, the proportion of households reporting a member with a problem with alcohol use, or the proportion of households using pesticides (and therefore eligible for a container) in each cluster, with clusters grouped by tertiles (table 3). There was no evidence of a change over time in the effectiveness of the containers (table 3, figure 3).

**Table 3:** Intervention effect modification by prespecified subgroup

	**Intervention**		**Control**		Relative rate ratio*(95% CI)	**p value for interaction**†
	Events/person-years	Incidence per 100 000 person-years	Events/person-years	Incidence per 100 000 person-years		
**Cluster historical rate of pesticide self-poisoning**						
Tertile 1	143/64 622	221·3	130/53 398	243·5	··	··
Tertile 2	169/64 851	260·6	176/65 378	269·2	··	··
Tertile 3	299/78 853	379·2	335/82 765	404·8	0·98 (0·82–1·18)	0·86
**Proportion of households in cluster reporting a member with a problem with alcohol use**						
Tertile 1 (0–23%)	206/73 238	281·3	179/67 892	263·7	··	··
Tertile 2 (23–28%)	175/61 169	286·1	240/68 856	348·6	··	··
Tertile 3 (28–45%)	230/73 920	311·2	222/64 794	342·6	0·93 (0·78–1·11)	0·41
**Proportion of households in cluster eligible for a lockable box**						
Tertile 1 (20–77%)	178/64 332	276·7	261/86 512	301·7	··	··
Tertile 2 (77–86%)	208/75 597	275·1	235/73 119	321·4	··	··
Tertile 3 (86–98%)	225/68 397	329·0	145/41 910	346·0	1·01 (0·84–1·21)	0·93
**Time since distribution of lockable boxes to cluster**						
Year 1	238/69 442	342·7	247/67 180	367·7	··	··
Year 2	192/69 442	276·5	195/67 180	290·3	··	··
Year 3	181/69 442	260·6	199/67 180	296·2	0·97 (0·85–1·11)	0·69

* Relative rate ratio estimating any trend in the magnitude of intervention effect per tertile increase, adjusted for person-years of follow-up of people in households eligible for a lockable box, and rate of previous pesticide self-poisoning in the cluster.

† p values are from likelihood ratio tests.

In the end of study demographic survey of 13 999 (26%) households in four bands, 147 episodes of pesticide self-poisoning were reported by households. As a quality-control exercise, we attempted to match these cases to our prospective follow-up. Only six (4%, three in each group) of these cases could not be matched and were judged as being missed by the follow-up because of presentation to hospitals outside the study area and residents who maintained multiple addresses. Notably, our prospective follow-up identified 634 cases of pesticide self-poisoning in these four bands, about four times more than self-reported by households.

The design effect for the analysis of the primary outcome was estimated as the ratio of the squared standard errors for the intervention effect estimates from the random effect Poisson regression model and the standard Poisson regression model, both including covariates for the person-years of follow-up contributed by individuals in households eligible for a lockable container, and historical rate of pesticide self-poisoning in the cluster. This gave an estimate of the design effect of 1·75, identical to that allowed for in the sample size calculation.

We found no evidence that the intervention caused a switch from pesticide self-poisoning to other forms of self-harm. The incidence of fatal and non-fatal self-harm events involving all methods in people aged 14 years or older did not differ between the intervention and control groups (table 3). There was also no evidence of switching from pesticide self-poisoning to other forms of self-poisoning (non-fatal or fatal). We noted no substantial increase in other common forms of self-harm (hanging, burning, cutting; table 4).

**Table 4:** Methods of self-harm (fatal and non-fatal) other than self-poisoning in individuals aged 14 years or older

	**Number of events**	**Incidence per 100 000 person-years**	**Number of fatal events (%)**
**Hanging**			
Intervention	36	17·3	26 (72%)
Control	21	10·4	14 (67%)
**Self-cutting**			
Intervention	18	8·6	0
Control	11	5·5	1 (9%)
**Self-burning**			
Intervention	4	1·9	2 (50%)
Control	8	4·0	2 (25%)
Other*			
Intervention	5	2·4	4 (80%)
Control	7	3·5	4 (57%)

* Includes jumping in front of train (n=5) or road vehicle (n=1), drowning (n=3), other self injury (n=2), and not specified (n=1).

Pesticide poisoning in children younger than 14 years was rare, with only 18 cases in the intervention group and 15 cases in the control group recorded during the 3 years of follow-up (incidence 21·8 per 100 000 person-years of follow-up in the control group *vs* 25·0 per 100 000 in the intervention group). Similarly, unintentional and occupational poisoning requiring presentation to hospital was uncommon, with only four cases in the intervention group and nine cases in the control group during the 3 years of follow-up.

## Discussion

Means restriction is an effective approach to suicide prevention.^29^ Small pilot studies have shown that improved pesticide storage, with household or community storage systems, is acceptable to farming communities and possibly effective.^21^, ^22^ As a result, improved storage has been widely promoted by civil society, industry, and international multilateral agencies. However, results of this large pragmatic cluster-randomised controlled trial showed that improved household storage of pesticides did not reduce the incidence of pesticide self-poisoning during 3 years of follow-up.

Improved storage is a very active form of prevention, requiring persistent and lifelong effort by individuals and families to store pesticides away after purchase and use, to keep key(s) hidden, to replace locks when damaged or the key lost, and to replace damaged containers. Our analysis of five sentinel villages showed that self-reported use of locked containers was 72% by 1 year; by 3 years, surveys of households in the intervention group showed that 53% were still using the container and locking it. Repeated reminders to households might sustain effective use; this approach has been used in some previous pilot studies. However, this strategy will not be sustainable if expanded to generalised public health use. For this reason, other than the five sentinel villages, we did not access clusters after the installation check 2 weeks after distribution, relying on community posters and routine farming meetings to encourage use. However, a failure of reminders does not seem to have been pivotal because we found no evidence that the containers were more effective in the first year after distribution compared with the third year.

To reduce contamination across the study, we ensured that the containers were installed in the ground to reduce the risk of them being sold on to families in non-intervention clusters;^25^ the low number of households in the control areas with pesticides locked away in non-study containers in the end of study demographic survey suggests that the idea of locking up pesticides did not effectively pass from intervention to control areas. We took account of possible contamination, and non-use of containers in intervention clusters, by substantially increasing the size of the study. The number of cases detected was higher than expected in our initial power calculations (crude population incidence 871 per 100 000 over 3 years *vs* 525 per 100 000 over 3 years estimated in our power calculations), further increasing the study power, thus ensuring that the study had sufficient power to detect an effect of magnitude important to public health.

Introduction of means restriction might result in method substitution, possibly to methods of suicide with higher case fatality (such as hanging). We found no evidence of increased rates of self-harm from other methods or non-pesticide forms of self-poisoning. The provision of the lockable container did not appear to increase the risk of self-harm because of farmers bringing pesticides into the home compound. There was a small, non-significant increase in the rate of fatal self-harm in the intervention clusters. It remains possible that provision of household storage containers might increase the incidence of higher lethality methods such as hanging, but the incidence of this method remained low in this study.

Our study has some limitations. We might have missed some non-fatal self-harm cases by using peripheral hospitals as the primary source of data. However, previous work in the North Central Province has suggested that nearly all cases of self-harm in rural areas are brought to small rural hospitals.^30^ Furthermore, we have no evidence that there was differential hospital presentation between the two groups of the study and the cases substantially exceeded the predicted rate.

The research staff involved in outcome ascertainment were, as far as possible, kept masked to the trial allocation of each participant's village (cluster) of residence. However, this was not absolute because when presenting to hospital a few patients reported taking pesticides from a study container. To control this possible source of bias, we maintained multiple separate study teams with limited interactions.

This study only assessed the use of household-based storage containers, not community-based locker storage systems as proposed by some advocates.^23^ However, it seems likely that compliance with a central community locker would be poorer in real life than compliance with household storage, since the former will require farmers to go away from their fields, towards the town centre to obtain pesticides.

Prevention of pesticide suicides is multi-layered, requiring work at individual, community, and population levels.^2^, ^31^ Means restriction is a key element of suicide prevention strategies, working at the community and regulatory levels.^29^ Unfortunately, our findings show that improved household storage of pesticides is unlikely to be effective. Individual level interventions will be administrative and active, requiring active choices by individuals to lower their risk. Improving medical care will prevent some deaths.^31^ However, this approach is difficult for the most commonly used pesticides because of the absence of new effective therapies, and the scarcity of human resources and hospital facilities in regions with many patients.^18^, ^19^ Another strategy that has been assessed is the addition of anti-emetics to paraquat formulations; however, this intervention only had a modest effect.^32^ Commentary on the study suggested that this was the wrong approach and that restricting access through regulation was likely to be more effective.^33^

Other interventions at the regulatory level, including replacing highly hazardous pesticides in agricultural practice with integrated pest management and alternative less hazardous pesticides, has major beneficial effects on both pesticide suicides and total suicides.^34^ Such regulatory action in Sri Lanka, for example, has resulted in a 75% reduction in total suicides with an estimated 93 000 lives saved over 20 years and little if any effect on agricultural yield.^35^, ^36^, ^37^ Similar data have come from South Korea^38^ and Bangladesh.^39^ Some data from China have shown the important role of urbanisation in the reduced suicide rate reported in this country.^40^ Urbanisation is occurring worldwide, but its effect is likely to reduce the population at risk and enhance means restriction interventions. Clearly, policy makers worldwide can best focus their means restriction efforts on working with agricultural colleagues to follow the Code of Conduct on Pesticide Management, assess the need for highly hazardous pesticides, and remove all that are not essential.^40^
