# Department of Error

**DOI:** 10.1016/S0140-6736(17)33089-1

**Published:** 2017-12-09

*Pearson M, Metcalfe C, Jayamanne S, et al. Effectiveness of household lockable pesticide storage to reduce pesticide self-poisoning in rural Asia: a community-based, cluster-randomised controlled trial.* Lancet *2017; **390:** 1863–72—*In this Article (published online first on Aug 11, 2017), Martin Wilks (Swiss Centre for Applied Human Toxicology, University of Basel, Switzerland) should have been cited in the Data Monitoring Committee. This correction has been made to the online version as of Dec 7, 2017.

