# Supplementary material for: Effectiveness of household lockable pesticide storage to reduce pesticide self-poisoning in rural Asia: a community-based, cluster-randomised controlled trial
Source: Lancet. 2017 Oct 21;390(10105):1863–72. doi: 10.1016/S0140-6736(17)31961-X (PMC5655546; doi:10.1016/S0140-6736(17)31961-X)

THE LANCET

Supplementary appendix

This appendix formed part of the original submission and has been peer reviewed. We post it as supplied by the authors.

Supplement to: Pearson M, Metcalfe C, Jayamanne S, et al. Effectiveness of household lockable pesticide storage to reduce pesticide self-poisoning in rural Asia: a community-based, cluster-randomised controlled trial. *Lancet* 2017; published online Aug 11. http://dx.doi.org/10.1016/S0140-6736(17)31961-X.

Supplementary information

Table S1. Primary analysis repeated under different sensitivity scenarios*

	n events	Rate / 10^5	RR	(95% CI)	p-value
Primary analysis (from Table 2)					
Intervention	611	293.3	0.93	(0.80, 1.08)	0.33
Comparison	641	318.0			
1. Utilisation study clusters excluded					
Intervention	528	282.8	0.92	(0.79, 1.07)	0.29
Comparison	641	318.0			
2. Date of box distribution per cluster					
Intervention	612	293.8	0.93	(0.80, 1.08)	0.34
Comparison	641	318.0			
3. Events classified by expert committee excluded					
Intervention	607	291.4	0.93	(0.81, 1.08)	0.36
Comparison	635	315.1			

*

1. After dropping the five intervention clusters (sentinel villages) in which there was greater researcher presence while the container utilization study was performed.
2. Instead of using the start of box distribution in a band (logistic areas – see randomisation and masking) as the start of three-year follow-up for all clusters within that band, this analysis used the end of box distribution in that cluster for the start of follow-up.
3. This analysis excluded all cases that required co-investigator review of means and the intent.

Lockable pesticide container: Pesticide container used in the study. See Pearson et al. (2011) for further information on the container

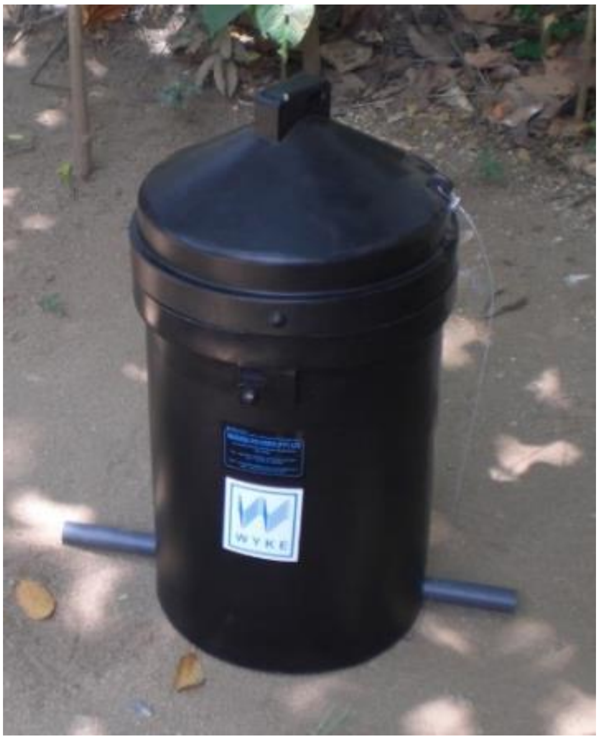

Supplement: Supplementary appendix [file mmc1.pdf]
